# Chemotherapeutic Interventions Against Tuberculosis

**DOI:** 10.3390/ph5070690

**Published:** 2012-06-28

**Authors:** Neeraj Shakya, Gaurav Garg, Babita Agrawal, Rakesh Kumar

**Affiliations:** 1 Department of Laboratory Medicine and Pathology, 728-Heritage Medical Research Center, Faculty of Medicine and Dentistry, University of Alberta, Edmonton, AB T6G 2S2, Canada; 2 Department of Pharmacy, Mangalayatan University, Beswan, Aligarh 202 145, India; 3 Department of Surgery, 728-Heritage Medical Research Center, Faculty of Medicine and Dentistry, University of Alberta, Edmonton, AB T6G 2S2, Canada

**Keywords:** tuberculosis chemotherapy, mycobacterium, drug-resistant tuberculosis, emerging TB drugs

## Abstract

Tuberculosis is the second leading cause of infectious deaths globally. Many effective conventional antimycobacterial drugs have been available, however, emergence of multidrug-resistant tuberculosis (MDR-TB) and extensively drug-resistant tuberculosis (XDR-TB) has overshadowed the effectiveness of the current first and second line drugs. Further, currently available agents are complicated by serious side effects, drug interactions and long-term administration. This has prompted urgent research efforts in the discovery and development of new anti-tuberculosis agent(s). Several families of compounds are currently being explored for the treatment of tuberculosis. This review article presents an account of the existing chemotherapeutics and highlights the therapeutic potential of emerging molecules that are at different stages of development for the management of tuberculosis disease.

## 1. Introduction

In 1905, Robert Koch, a German physician, was awarded the Nobel Prize for his milestone discovery of *Mycobacterium tuberculosis* (*Mtb*), the bacillus of tuberculosis (TB). Despite his groundbreaking discovery, it took more than half a century to find a cure against the bacilli to save millions of human lives. The death of Koch in 1910 prevented him from witnessing the life-saving consequences of his pioneering research. Among these was the first antibiotic for tuberculosis patients, streptomycin, a natural compound. However, the requirement of intravenous administration of streptomycin and development of resistance to it soon necessitated the need of next generation of antibiotics against *Mtb*. Many antimycobacterial drugs have been discovered since then, classified as first-line and second line drugs. Recent emergence of multidrug-resistant tuberculosis (MDR-TB) and extensively drug-resistant tuberculosis (XDR-TB) [1], have seriously compromised the usefulness of these current first and second line drugs, once again creating an urgent need for newer, safer and more effective anti-tuberulosis treatments. Adding to this crisis is the limited use of second line drugs for MDR-TB and XDR-TB due to their toxicity and serious side effects. Moreover, recently, totally drug-resistant tuberculosis (TDR-TB) has emerged which is resistant to a wider range of drugs than XDR-TB. Cases of TDR-TB have been reported in several countries including Italy, Iran and India [2].

Bacillus Calmette Guerin (BCG) vaccine, an attenuated strain of *M. bovis* reliably protects only newborns against *Mtb* but is ineffective in adult pulmonary TB. The vaccination may also lead to TB-like infection in immunocompromised people [3].

The increasing incidence of MDR-TB, XDR-TB, and TB-HIV coinfection have raised the alarm for the discovery and development of novel anti-tuberculosis agent(s) that do not possess cross-resistance with current antimycobacterial drugs and have minimal toxicity. This article summarizes the features of current anti-tuberculosis drugs and the pharmacological properties of novel compounds that are in the process of development for antimycobacterial therapy.

## 2. Current Anti-Tuberculosis Drugs

### 2.1. First Line Drugs

**Figure 1:**
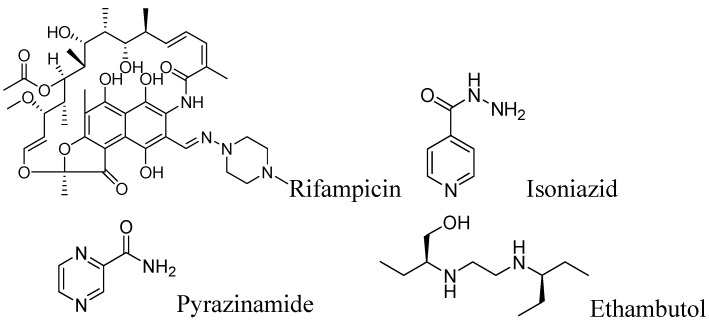
First line anti-tuberculosis drugs.

First line anti-tuberculosis drugs include rifampicin, isoniazid, pyrazinamide and ethambutol (Figure 1).

#### 2.1.1. Rifampicin

This drug was discovered in 1966. It possesses very potent *in vitro* activity against *Mtb* with an MIC of 0.05–0.5 μg/mL. Rifampicin is highly active against Gram-positive bacteria including *Mtb. *Unlike many other antibiotics, it is lipid soluble, penetrates cell membranes and kills intracellular bacteria [4]. It acts by the inhibition of DNA-dependent RNA polymerase in bacterial cells by binding to its β-subunit, thus preventing transcription of RNA and subsequent translation of proteins [5,6]. A daily regimen of 10 mg/kg (up to 600 mg/day) orally or an intermittent regimen of 10 mg/kg (up to 600 mg/day) orally, are effective [7]. However, *Mtb* quickly develops resistance to rifampicin hence the drug is recommended to be used in combination with other antibiotics. Most of the *Mtb* clinical isolates resistant to rifampicin show mutations in the *rpoB* gene that encodes the *β*-subunit of RNA polymerase. These mutations cause conformational changes in the polymerase that result in a low affinity for the drug rendering it ineffective [8]. The side effects include hepatitis with elevation of bile and bilirubin, anaemia, leucopenia, thrombocytopenia, bleeding, fever, eosinophilia, leucopenia, thrombocytopenia, purpura, haemolysis and nephrotoxicity [9]. Interestingly, no serious side effects have been observed in breastfed infants during rifampicin therapy [10,11]. The drugs for possible interactions with rifampicin include 4-aminosalicylic acid (PAS), HIV protease inhibitors, warfarin, oral contraceptives, cyclosporine, itraconazole, digoxin, verapamil, nifedipine, simvastatin, midazolam, clarithromycin, lorazepam atorvastatin, antiretroviral agents, rosiglitazone/pioglitazone, celecoxib, caspofungin [12].

#### 2.1.2. Isoniazid

Isoniazid (INH) was discovered in 1952. It acts as a bactericidal agent with an MIC of 0.01–0.2 μg/mL for fast replicating mycobacteria [13]. It is bacteriostatic to slow-growing or non-dividing mycobacteria like *Mtb* and therefore, is used to treat latent tuberculosis. Isoniazid is a actually a prodrug and is activated by the mycobacterial enzyme catalase-peroxidase (KatG), which catalyzes the formation of the isonicotinic acyl-NADH complex. Subsequently, this complex binds to the enoyl-acyl carrier protein reductase InhA, and then blocks the natural substrate enoyl-AcpM and fatty acid synthase. This results in inhibition of mycolic acid synthesis which is an essential component in the formation of the mycobacterial cell wall [14,15]. Resistance to isoniazid occurs due to mutations in several genes, including *katG*, *ahpC*, *inhA*, *kasA* and *ndh*. In adults, the recommended daily dose of INH is 5 mg/kg/day (max 300 mg daily). For intermittent dosing (twice or three times/week), 19–15 mg/kg/day (max 900 mg/day) is used. The recommended dose for children is 8 to 12 mg/kg/day [7,16]. INH is metabolized in the liver and its metabolites are excreted in the urine [17]. INH chronic toxicity affects the liver, haematologic- and peripheral nervous systems resulting in acute hepatitis, peripheral neuropathy and haemolytic anaemia [18].

#### 2.1.3. Pyrazinamide

Pyrazinamide (PZA) was discovered in 1952. It is mainly bacteriostatic but can be bactericidal for replicating *Mtb*. It possesses an MIC of 20–100 μg/mL. When used as part of combination therapy, PZA accelerates the sterilizing effect of INH and rifampin [19]. This has enabled reductions in the duration of treatment for susceptible *M. tuberculosis* isolates from nine to six months and for this reason is used in the first two months of treatment [20]. PZA is also effective for the treatment of tuberculous meningitis [21]. Like isoniazid, PZA is a prodrug. In acidic conditions, the enzyme pyrazinamidase (present in *Mtb*), converts it to the active form, pyrazinoic acid which subsequently inhibits the enzyme fatty acid synthase (FAS) I, required by the bacterium to synthesize fatty acids [22,23,24]. Mutations of the pyrazinamidase gene (*pncA*) are responsible for PZA resistance in *Mtb *[25]*.* Most alterations occur in a 561 bp region of the open reading frame or in an 82 bp region of its putative promoter [26,27].

The recommended dose of PZA is 20–25 mg/kg daily or 30–40 mg/kg three times a week [7]. Pyrazinamide is metabolized by the liver and the metabolic products are excreted by the kidneys [28]. Some common side effects of PZA include skin rash, nausea, vomiting, hepatotoxicity, anorexia, hyperuricemia, sideroblastic anemia, dysuria, joint pains (arthralgia), urticaria, pruritus, malaise, interstitial nephritis, porphyria and fever [29].

#### 2.1.4. Ethambutol

This drug was discovered in 1961. Ethambutol (EMB) is a bacteriostatic drug against actively growing mycobacteria. It blocks formation of the cell wall of *Mtb *by inhibiting the enzyme arabinosyl transferase involved in the synthesis of arabinogalactan. Arabinogalactan is an essential component in the formation of the mycolyl-arabinogalactan-peptidoglycan complex of the *Mtb* cell wall [30,31]. Mutation in gene *embB* is responsible for resistance to ethambutol [30].

Ethambutol is well absorbed in the gastrointestinal tract, and is efficiently distributed in body tissues and fluids. Fifty percent of the given dose is excreted unchanged in urine [32]. Ethambutol is used at 15–25 mg/Kg once daily dose for 6–8 weeks concurrent with isoniazid therapy [33]. Adverse effects of EMB include peripheral neuropathy, red-green color blindness, arthralgia, hyperuricaemia and optic neuritis [34].

### 2.2. Second Line Drugs

The available second-line TB drugs can be classified as: (1) polypeptides (e.g., capreomycin); (2) aminoglycosides: (e.g., amikacin); (3) oxazolidinone (e.g., cycloserine); (4) thioamides (e.g., ethionamide); (5) fluoroquinolones (e.g., ciprofloxacin); (6) *p*-aminosalicylic acid (PAS or P). Some of the second line drugs are summarized in Table 1.

**Table 1:** Common second line drugs [35].

Drug (Discovery) MIC values *	Structure	Daily dose (Max. dose) Route	Adverse effects	Mode of action
Capreomycin (1963) MIC 1.25–2.5 μg/mL [ 36,37]	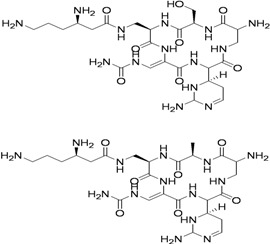	15–30 mg/kg (1 g) IM or IV	Auditory, vestibular, and renal toxicity	Inhibits protein synthesis (binds to ribosomal subunit 16S and 23S rRNA) [38]
Amikacin (1972) MIC 4–8 μg/mL [39]	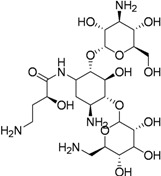	15–30 mg/kg (1 g) IM or IV	Same as capreomycin	Inhibits protein synthesis (binds to the bacterial 30S ribosome)
Kanamycin (1957) MIC 1–8 μg/mL	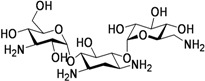	15–30 mg/kg (1 g) IM or IV	Same as Capreomycin	Inhibits protein synthesis via S12 ribosomal protein & 16 S RNA.
Streptomycin (1944) MIC 2–8 μg/mL	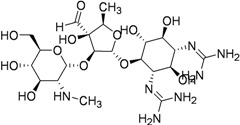	15–40 mg/kg (1 g) IM	Renal, ophthalmic and respiratory toxicity	Same as kanamycin
Cycloserine (1952) MIC 5–20 μg/mL	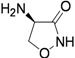	15–20 mg/kg (1 g) Oral	Psychosis, Rashes, Convulsions Depression	Inhibition of peptidoglycan synthesis (D-alanine racemase)
Ethionamide (1956) MIC 0.6–2.5 μg/mL	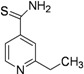	15–20 mg/kg (1 g) Oral	GI upset Hepatotoxicity Hypersensitivity	Inhibition of mycolic acid synthesis
Clofazimine (1954) MIC 0.12–0.24 μg/mL [40]	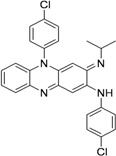	100–300 mg/day Oral	Eosinophilic enteritis, GI irritation, discoloration of the skin (upon sun exposure)	Inhibits bacterial proliferation by binding to the guanine bases of bacterial DNA
Levofloxacin (1992) MIC 0.50 to 0.75 μg/mL [41]	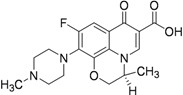	500 mg/day Oral	GI upset Dizziness Headache Hypersensitivity Restlessness	Inhibition of DNA replication and transcription by inhibiting DNA gyrase
Ofloxacin (1980) Oral, MIC 0.12–2 μg/mL [42]	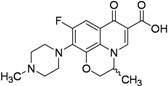	600–800 mg/day	Same as for levofloxacin	Same as for levofloxacin
Ciprofloxacin (1960s) MIC 0.4 to 6.2 μg/mL [43]	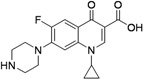	750–1,500 mg/day Oral	Same as for levofloxacin	Same as for levofloxacin
PAS (1946) MIC 1–8 μg/mL	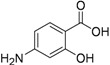	150 mg/kg (16 g) Oral	Same as for ethionamide, Sodium load	Inhibition of folic acid and iron metabolism (unknown target)

* MICs (wherever not referenced) are based on Inderlied and Salfinger [44]. IM, intramuscular; IV, intravenous.

## 3. Drug Discovery Program

This section includes early stage drug discovery, molecules in development, molecules at the pre-clinical stage, molecules in phase I trials, molecules in phase II trials and molecules in phase III trials.

### 3.1. Early Stage Drug Discovery

After a long pause, the last decade proved to be a golden era in the hunt for new tuberculosis drug(s). Tremendous efforts and high priority research are underway for finding better drugs to combat wild-type and drug-resistant *Mtb*. Since the last decade, the private sector and government agencies have participated in the fight against this devastating disease. Apart from major financial contributions by corporations, basic and semi-applied researchers are also continuing to make significant progress, despite facing financial constraints. The following section describes some representative examples of different classes of molecules from early stage screening studies carried out in the last decade by these groups.

#### 3.1.1. Nucleosides

The nucleoside class of compounds are well known for their antiviral and anticancer properties. They can be classified as pyrimidine or purine nucleosides.

##### 3.1.1.1. Pyrimidine Nucleosides

**Figure 2:**
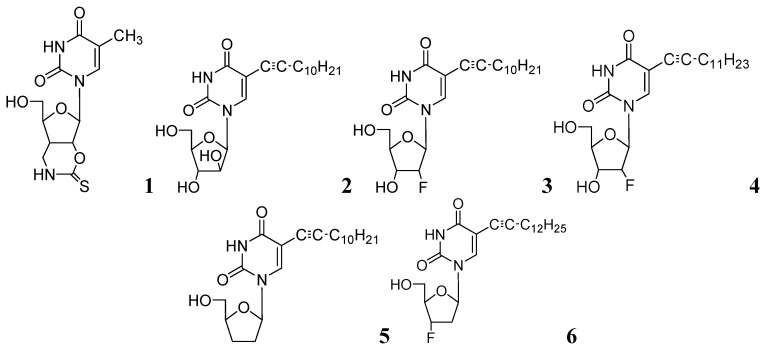
Pyrimidine nucleosides as anti-tuberculosis agents.

In early 2000s, Vanheusden *et al*. reported modified nucleoside and nucleotide derivatives as inhibitors of a mycobacterial enzyme thymidine monophosphate kinase (TMPKmt). In 2004, they reported a series of bicyclic analogues of thymidine [45] where compound **1** (Figure 2) demonstrated a Ki of 3.5 μM for TMPKmt with a good selectivity index (SI 200) over its human counterpart TMPKh. In these studies, however, only enzyme inhibition was reported and inhibition of mycobacterial replication was not demonstrated.

The complete genome sequence of *Mycobacterium tuberculosis *has been determined [46], which identified many of the genes required for encoding enzymes involved in nucleic acid synthesis, and pyrimidine and purine biosynthesis. We hypothesized that modified nucleosides could target several enzymes involved in RNA and DNA metabolism and were the first to investigate and demonstrate potent antimycobacterial activity of 5-substituted pyrimidine nucleoside analogs [47]. The microplate alamar blue assay (MABA) [48] was used to evaluate the antimycobacterial activity of test nucleosides. We observed that the most potent TMPKmt inhibitors reported earlier [49,50,51] did not show antituberculosis activity against mycobacterial replication as determined by MABA assay [47].

Since our initial report in 2005, we (Kumar and colleagues) have made significant contributions in the investigation of pyrimidine nucleosides as new classes of anti-tuberculosis agents. We designed, synthesized and examined a variety of known and unknown pyrimidine nucleosides substituted/unsubstituted at 2-, 4-, 5- and/or 6- positions of the base, and containing various deoxyribose, ribose, arabinose, dideoxyribose and acyclic sugar moieties. We found that 5-alkynyl substituted pyrimidine nucleosides demonstrated the most potent activity against mycobacteria [52,53]. The MIC_90_ exhibited by compounds of this series (**2**, **3** and **4**, Figure 2) was in the range of 1–5 μg/mL) against *Mtb *H37Ra. These compounds were also found to retain sensitivity against an RMP-resistant strain of *Mtb *H37Rv (American Type Culture Collection [ATCC] 35838, resistant to RMP at 2 μg/mL) at similar concentrations. Subsequently, we reported a series of 5-acetylenic derivatives with 2',3-dideoxyuridine, and 3'-fluoro-2',3'-dideoxyuridine. Compound **5** (among 2',3'-dideoxyuridine series) and compound **6** (among 3'-fluoro-2',3'-dideoxyuridine series) exhibited excellent activity against wild-type *Mtb* H_37_Ra (MIC 1–2 μg/mL) and a rifampicin-resistant H37Rv strain (ATCC 35838, resistant to RMP at 2 μg/mL) of *Mtb* [54] (Figure 2).

**Figure 3:**
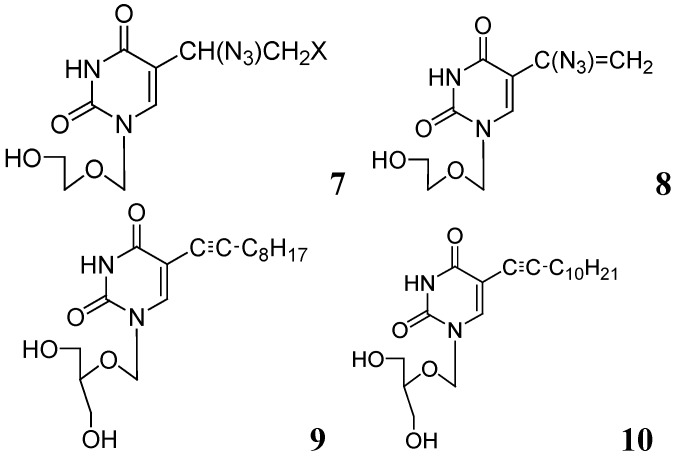
Acyclic pyrimidine nucleosides as anti-tuberculosis agents.

In further studies, we also investigated anti-tuberculosis activities of several 5-substituted acyclic pyrimidine nucleosides against *Mtb* H37Ra, *M. bovis*, and *M. avium*. In this study, **7–10** were moderately active against these mycobacteria [55] (Figure 3).

In recent studies, we synthesized and investigated various 2'- or 3'-halogeno derivatives of pyrimidine nucleosides containing uracil, 5-fluorouracil, and a thymine base [56]. In this class, compound **11** was found to be the most effective antituberculosis agent in the *in vitro* assays against wild-type *Mtb *strain (H37Ra, MIC_50_ = 1 μg/mL), and drug-resistant (H37Rv) strains of Mtb (RMP-resistant and INH-resistant, MIC_50_ = 1–2 μg/mL). The antimycobacterial effect of the most potent compounds was also determined against intracellular mycobacteria in a human monocytic cell-line (THP-1) infected with the *Mtb* H37Ra strain [57]. Interestingly, compound **11** demonstrated slightly better activity against intramacrophagic mycobacteria than extracellular mycobacteria. In contrast, pyrimidine nucleosides possessing a 5-fluorouracil base were weak inhibitors of *Mtb *H37Ra.

In the same year our group reported antimycobacterial effects of several 5-alkyl- and 5-alkynyl-furanopyrimidines and related 2'-deoxynucleosides. Compounds with 5-arylalkynyl substituents displayed potent *in vitro* antitubercular activity against *M. bovis* and *Mtb* (MIC 0.5–5 μg/mL). We selected compound **12** to test its potency in a mouse model (BALB/c) of *Mtb* (H37Ra) infection. At a dose of 50 mg/kg for 5 weeks, statistically significant reduction in mycobacterial load was observed in lungs, livers and spleens of the treated mice. This is the first evidence of antimycobacterial potential of 5-substituted pyrimidine nucleosides in an animal model as a potential new class of antituberculosis agents [58].

**Figure 4:**
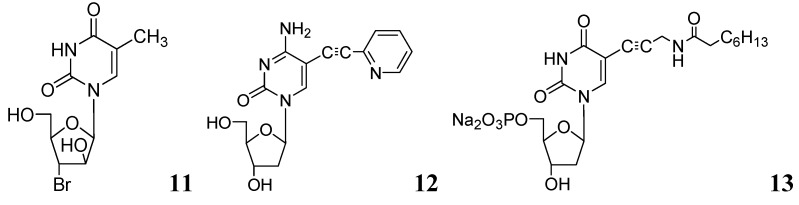
Some recent pyrimidine nucleosides as anti-tuberculosis agents.

Kogler *et al*. reported a series of 5-substituted -2'-deoxyuridine monophosphate analogs as potential inhibitors of mycobacterial flavin-dependent thymidylate synthase (ThyX). Compound **13** displayed selective inhibition of ThyX (IC_50_ 0.91 μM) but not against the classical mycobacterial thymidylate synthase (ThyA, IC_50_ > 50 μM) [59] (Figure 4).

##### 3.1.1.2. Purine Nucleosides

Somu *et al*. reported a purine nucleoside analog **14** (MIC_99_ = 0.19 μM) as an inhibitor of siderophore biosynthesis in *Mtb* under iron-limiting conditions. The authors mentioned that the activity of **14** was due to inhibition of the adenylate-forming enzyme MbtA, which is involved in biosynthesis of the mycobactins [60].

Triazole derivatives of 5'-*O*-[*N*-(salicyl)sulfamoyl]adenosine have been investigated as inhibitors of aryl acid adenylating enzymes (AAAE) involved in siderophore biosynthesis by *Mtb *H37Rv. Enzyme assays were performed at 37 °C with recombinant MbtA expressed in *E. coli*. Compound **15** (MIC 3.13 μM) was reported as the best candidate [61].

**Figure 5:**
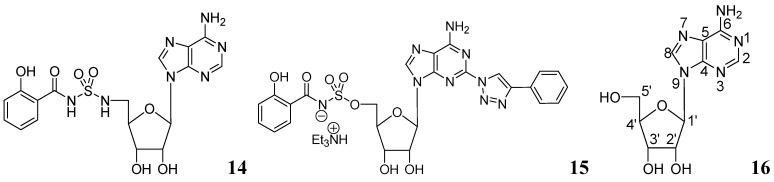
Purine nucleosides as anti-tuberculosis agents.

Adenosine (Ado) kinase is a purine salvage enzyme that phosphorylates adenosine to adenosine-monophosphate. A number of adenine nucleosides **16** have been evaluated as substrates and inhibitors of adenosine (Ado) kinase from *Mtb*. The best substrates were found to be 2-aza-adenosine, 8-aza-9-deazaadenosine and 2-fluoroadenosine, while the most potent compounds were N-1-benzyladenosine (Ki = 0.19 μM), 2-fluoroadenosine (Ki = 0.5 μM), 6-cyclopentyloxy purine riboside (Ki = 0.15 μM) and 7-iodo-7-deazaadenosine (Ki = 0.21 μM). Several of these adenosine analogs exhibited promising MICs [62] (Figure 5).

#### 3.1.2. Carbohydrates

Carbohydrates have been evaluated as antituberculosis agents for a long time. Some selected reports are summarized here. In 2005 bis-glycosylated diamino alcohols were reported by Tripathi *et al*., where their compound, **17**, showed moderate activity against *Mtb *H37Ra and against *Mtb* H37Rv. This compound was also active against an MDR strain and showed mild protection in mice at 25 mg/Kg dose [63].

Derivatives of stachyose were reported by Chiba *et al*. The most active compound in the series against *Mtb* H37Rv was **18** (OCT359, MIC 3.13 μg/mL) which was also evaluated against various drug-sensitive and -resistant clinical isolates of *Mtb*. Interestingly, 25 clinical isolates of drug-resistant *Mtb* and 19 drug-sensitive *Mtb* were sensitive to OCT359 (MICs ranging from 3.13 to 25 μg/mL) [64].

**Figure 6:**
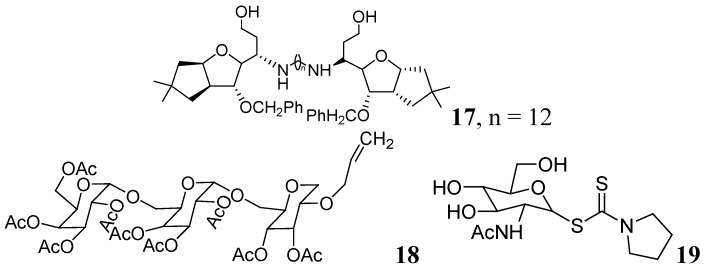
Carbohydrate derivatives as anti-tuberculosis agents.

Recently in this class, compound **19** (OCT313HK, Glc-NAc-PDTC) showed potent anti-tuberculosis activity against wild-type, and clinical isolates of *Mtb*, including MDR and XDR strains at similar concentrations (MIC 6.25–12.5 μg/mL) [65] (Figure 6).

#### 3.1.3. Heterocyclic Compounds

##### 3.1.3.1. Quinolines and Quinoxalines

Fluoroquinolones have been used as antibiotics (e.g., ciprofloxacin, levofloxacin, ofloxacin). Moxifloxacin and gatifloxacin from this class are in Phase III clinical trials for tuberculosis treatment.

Sriram *et al*. investigated a series of 7-substituted gatifloxacin derivatives. Their compound, **20**, was found to be the most active *in vitro* studies (MIC value 0.0125 μg/mL) against *Mtb* and MDR-TB. In an animal model **20** decreased the bacterial loads in the lung and spleen by 3.62- and 3.76-log10, respectively [66].

The same group investigated derivatives of ofloxacin (OFX). Compound **21** exhibited the most potent activity (MIC_99_ of 0.19 μM and 0.09 μM against *Mtb* and MDR-TB, respectively) and decreased bacterial loads (strain ATCC 35801) in lung and spleen by 1.91 and 2.91-log10, respectively, at 50 mg/kg dose in a mouse model [67].

**Figure 7:**
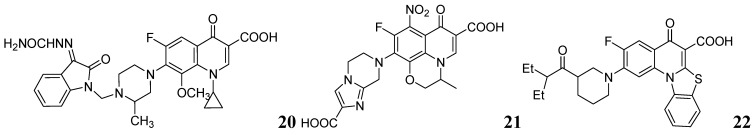
Fluoroquinolones as anti-tuberculosis agents.

Dinakaran *et al.* further reported derivatives of 2-(sub)-3-fluoro/nitro-5,12-dihydro-5-oxobenzothiazolo[3,2-a]quinoline-6-carboxylic acid. Among them, compound **22** displayed the most potent activity with MICs of 0.18 and 0.08 μM against *Mtb* and MDR-TB, respectively. In a mouse model of *Mtb* infection, **22** was effective at 50 mg/kg dose and reduced bacterial loads in lung and spleen tissues by 2.78 and 3.12-log10, respectively [68] (Figure 7).

In 2009, Senthilkumar *et al*. investigated 1-(substituted)-1,4-dihydro-6-nitro-4-oxo-7-(sub-secondary amino)-quinoline-3-carboxylic acids. *In vitro*, their compound, **23**, exhibited MICs of 0.08 and 0.16 μM against *Mtb* and MDR-TB, respectively. *In vivo* studies revealed that **23** led to a significant reduction in bacterial load in lung and spleen at 50 mg/kg dose [69].

**Figure 8:**
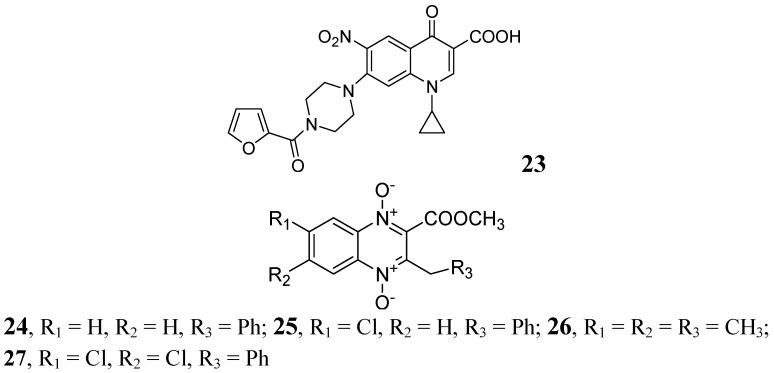
Quinoxaline-2-carboxylate 1,4-dioxide derivatives as anti-tuberculosis agents.

In 2005, Jaso *et al*. evaluated a series of 6(7)-substituted quinoxaline-2-carboxylate 1,4-dioxide derivatives against *Mtb*H37Rv. Their compounds **24**, (MIC 0.1 μg/mL) and **25**, (MIC 0.1 μg/mL) had good antituberculosis activity including intracellular bacteria (EC_90_ 0.15 μg/mL and 0.0005 μg/mL, respectively). Compounds **26** and **27** of the series were also active against drug-resistant strains of *Mtb* H37Rv with MICs of 0.39–1.56 and 3.13–12.5 μg/mL, respectively [70] (Figure 8).

**Figure 9:**
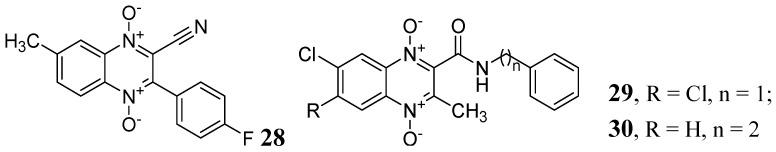
1,4-di-N-oxide-3-phenylquinoxalines as anti-tuberculosis agents.

A series of derivatives of 1,4-di-N-oxide-3-phenylquinoxaline was published by Vicente *et al.* against *Mtb *H37Rv. Their compound **28** was the most active in the series (MIC < 0.2 μg/mL) [71]. Ancizu *et al*. also described a series of 1,4-di-N-oxide derivatives of quinoline. Compounds **29** and **30** displayed the most significant inhibition of *Mtb* H37Rv with MICs < 0.2 μg/mL [72] (Figure 9).

**Figure 10:**
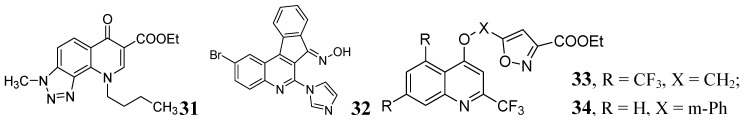
Some other quinoline derivatives as anti-tuberculosis agents.

Carta *et al*. published antituberculosis activity of 3-methyl-9-substituted-6-oxo-6,9-dihydro-3H-[1,2,3]-triazolo[4,5-h]quinolone-carboxylic acids and their esters against wild-type H37Rv and 11 clinically isolated strains of *Mtb*. The most potent compound in the series was **31** with MIC_90_ = 0.5 μg/mL [73] (Figure 10).

The indeno[2,1-c]quinoline derivatives described by Upadhayaya *et al*. were shown to be active with MICs in the range of 0.39–0.78 μg/mL. Ester derivatives in compound **32** retained the activity (MIC of <0.39 μg/mL) [74].

Lilienkampf *et al*. described quinoline compounds **33** and **34** with MICs of 0.77 μM and 0.95 μM, respectively, against the replicating *Mtb. *These two compounds also had activity against non-replicating persistent bacteria as well as RMP-, INH-, and streptomycin- resistant *Mtb *strains [75].

##### 3.1.3.2. Pyrimidine and Purines

**Figure 11:**
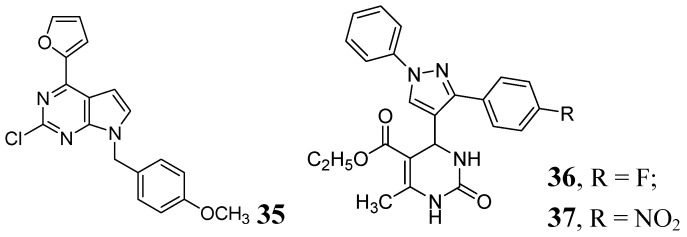
Pyrimidine and Purine analogs as anti-tuberculosis agents.

Various purine analogs were synthesized by Khoje *et al*. Compound **35** emerged as the most potent in the series (MIC 0.11 μM). The five most active compounds of the series were also evaluated against a panel of drug-resistant *Mtb* strains, where all of them retained activity. However, these compounds did not show good activity against non-replicating *Mtb* [76]. A series of dihydropyrimidines was examined by Trivedi *et al.* in 2010. Compounds **36** and **37** were the most potent in the series (MIC of 0.02 μg/mL) [77] (Figure 11).

##### 3.1.3.3. Azoles

**Figure 12:**
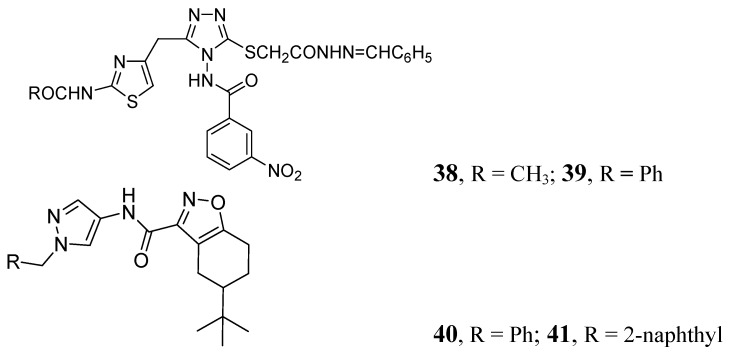
Azole analogs as anti-tuberculosis agents.

Many publications have emerged on azoles for anti-TB activity. Some good representatives of this class are described below. In this class, compounds **38** and **39** demonstrated MICs of 0.78 and 0.39 μM, against *Mtb* H37Rv respectively [78]. Pantothenate is a key precursor of coenzyme A and acyl carrier protein, essential for many intracellular processes. The PS pathway is not present in humans. Velaparthi *et al*. reported in 2008 compounds **40** and **41** (Figure 12) as the best inhibitors (IC_50_ of < 100 nM) [79] (Figure 12).

**Figure 13:**
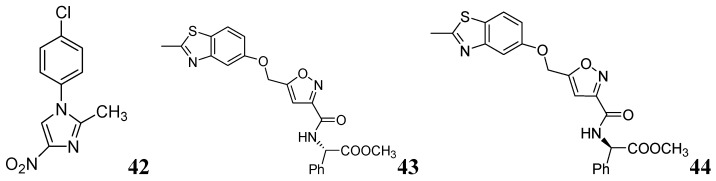
*N*-Aryl-*C*-nitroazoles as anti-tuberculosis agents.

*N*-Aryl-*C*-nitroazoles were investigated by Walczak *et al.* against H*37*Rv (ATCC 27294) using the MABA assay. Compound **42** exhibited an MIC of 0.39 μg/mL [80]. A series of 2-methylbenzothiazole derivatives was described by Huang *et al*. Compounds **43** and **44** were found to be potent inhibitors of replicating *Mtb* H37Rv (MIC 1.4 and 1.9 μM, respectively) [81] (Figure 13).

##### 3.1.3.4. Azines

**Figure 14:**
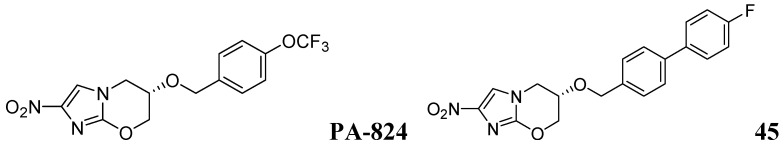
Azines as anti-tuberculosis agents.

Palmer *et al.* investigated antitubercular activity of biphenyl analogs of PA-824, which is currently in phase II clinical trial. The most active compound, **45**, had MICs of 0.015 and 1.4 μM in MABA and LORA assays, respectively. In a mouse model of acute *Mtb* infection, seven of the compounds showed substantially (>10-fold) improved efficacies and three of them were >200-fold more effective than PA-824 [82] (Figure 14).

##### 3.1.3.5. Pyridine hydrazides (INH analogs)

**Figure 15:**
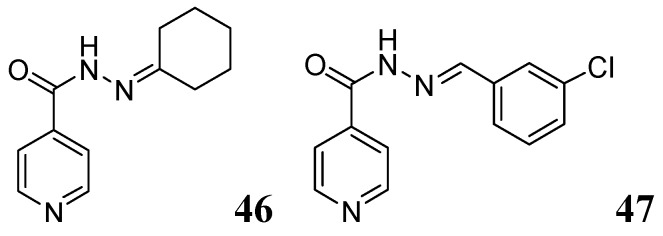
INH analogs as anti-tuberculosis agents.

Several Schiff bases of INH were synthesized by Hearn *et al.* that showed good *in vitro *activity and protected tuberculosis infection in mice. A representative cyclohexanone derivative, **46**, displayed an MIC of 0.03 μg/mL against *Mtb* H37Rv strain Erdman and exhibited reduction in mouse lung of 4.65 log CFU [83]. Lourenco *et al.* also reported a series of INH derivatives. Compound **47** exhibited significant *in vitro* activity (MIC 0.31 μg/mL) [84] (Figure 15).

##### 3.1.3.6. Miscellaneous

###### 3.1.3.6.1. Artemisinin Analog

**Figure 16:**
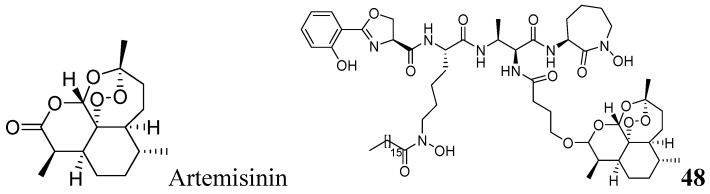
Artemisinin analog as anti-tuberculosis agents.

Artemisinin, commonly known as qinghaosu, is a natural sesquiterpene peroxide with a 1,2,4-trioxane nucleus, and is a highly active antimalarial agent. Miller *et al.* reported a mycobactin-artemisinin conjugate, **48**, with submicromolar activity against different clinical strains of tuberculosis [85] (Figure 16).

###### 3.1.3.6.2. Macrolides

**Figure 17:**
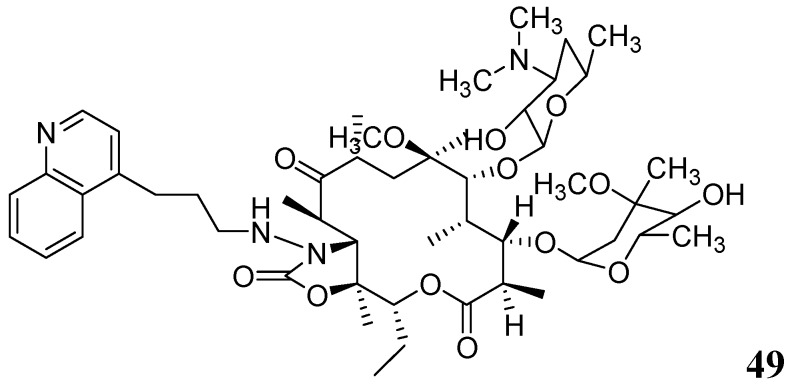
Macrolide as anti-tuberculosis agents.

Falzari *et al.* reported descladinose derivatives of macrolides and ketolides. Many compounds demonstrated submicromolar MICs against *Mtb*. Compound **49** (RU66252) emerged as a promising inhibitor with an MIC of 0.25 μM [86] (Figure 17).

###### 3.1.3.6.3. Thiolactomycin

**Figure 18:**
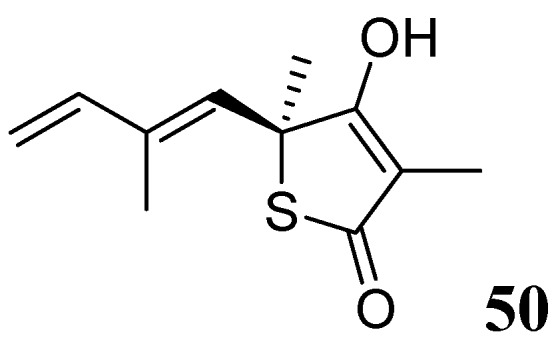
Structure of thiolactomycin.

Thiolactomycin (TLM), **50**, is a natural product isolated from *Nocardia *and *Streptomyces* species. TLM is an inhibitor of the *β*-ketoacyl-acyl carrier protein synthase (KAS) enzymes, which are part of the bacterial fatty acid synthase pathway. TLM has MIC of 62.5 μM against *Mtb* [87,88]. TLM also inhibits human FAS-I enzyme [89], however, its lower affinity (IC_50_ 100 μM) for this enzyme can make it worthy as a selective anti-tuberculosis agent [90] (Figure 18).

### 3.2. Molecules in Development

The following are compounds at various stages of preclinical and clinical development [91,92].

#### 3.2.1. Molecules at Pre-Clinical Stage

##### 3.2.1.1. CPZEN-45

**Figure 19:**
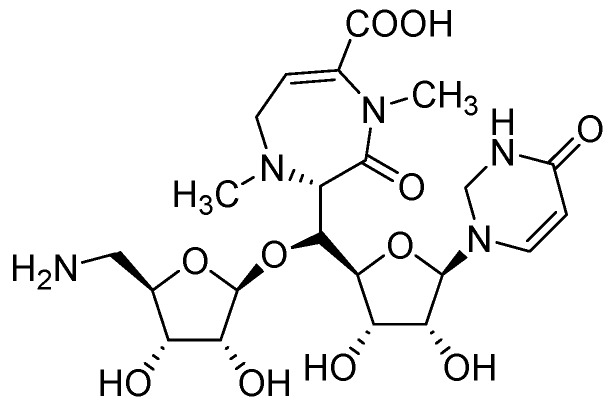
Structure of CPZEN-45.

CPZEN-45 (MIC of 1.56 μg/mL, *Mtb* H37Rv and 6.25 μg/mL, MDR strain of *Mtb*) is a nucleoside antibiotic produced by *Streptomyces* spp. CPZEN-45 is active against both replicating and on-replicating *Mtb in vitro*. It is also effective against both drug sensitive and extremely drug resistant (XDR) *Mtb* in a mouse model of acute tuberculosis with 1–1.5 log CFU reduction in the lungs. Its mode of action is not specified [93] (Figure 19).

##### 3.2.1.2. Quinolone DC-159a

**Figure 20:**
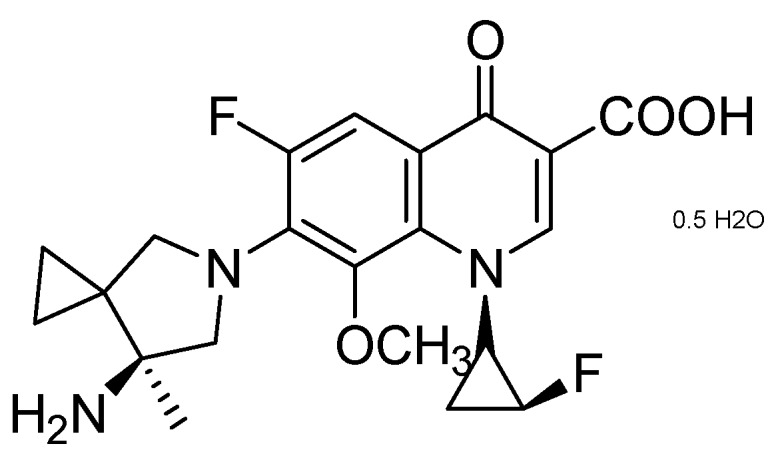
Structure of DC-159a.

DC-159a (MIC = 0.03 μg/mL) exhibited better early bactericidal activity (EBA) and higher log reduction of CFU in lungs against drug-susceptible, and quinolone-resistant (QR) MDR-TB, compared to that of moxifloxacin, gatifloxacin, levofloxacin and rifampicin. It acts by inhibiting DNA gyrase of wild-type and MDR-*Mtb*. In the QR MDR-TB infection model, it showed 2–3 times longer “mean survival days” which was superior to moxifloxacin, levofloxacin, INH and RMP (Figure 20).

##### 3.2.1.3. SQ-609

**Figure 21:**
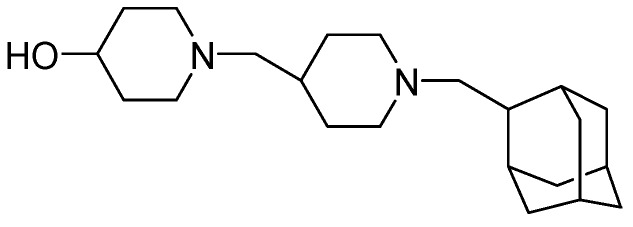
Structure of SQ609.

Sequella identified a promising candidate, SQ609, as the most potent among a new series of potential cell-wall inhibiting dipiperidines (MIC = 4 μg/mL). The precise mode of action of SQ 609 is unknown [94,95] (Figure 21).

##### 3.2.1.4. SQ-641

**Figure 22:**
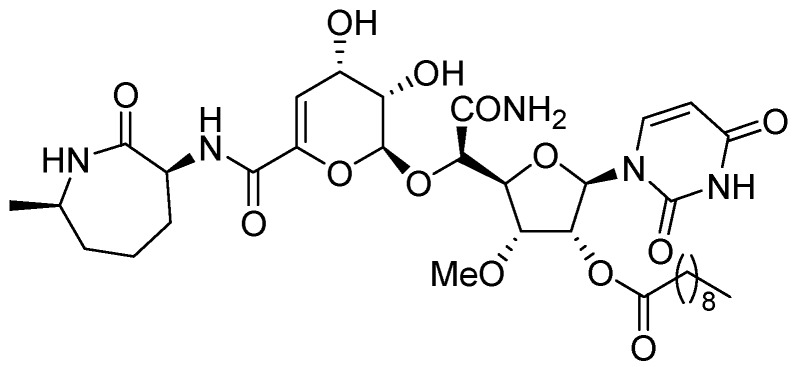
Structure of SQ-641.

The enzyme translocase 1 (TL1), which is absent in eukaryotic cells, is an essential enzyme in bacteria for the biosynthesis peptidoglycan in the cell wall. SQ-641, which targets TL1, possesses activity against MDR clinical strains of *Mtb *(MIC = 0.5 μg/mL). It has shown efficacy in a mouse model of chronic TB by reducing the CFU in lungs of infected mice by 1.0 to 1.5 log [91,94] (Figure 22).

##### 3.2.1.5. Benzothiazinone (BTZ-043)

**Figure 23:**
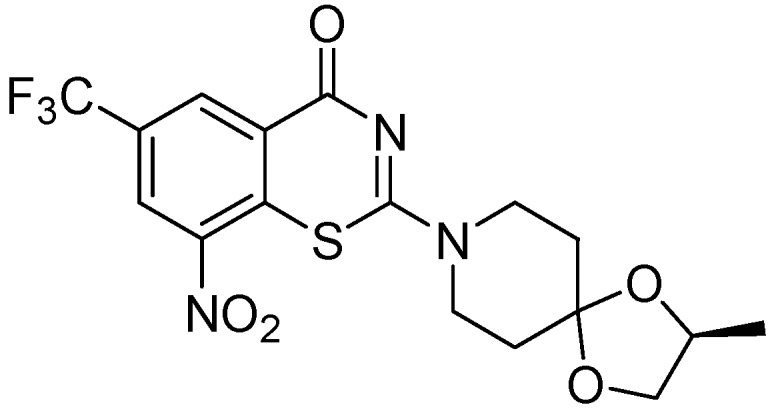
Structure of BTZ-043.

BTZ-043 is highly active against *Mtb *(MIC = 1–10 ng/mL) and other actinobacteria. BTZ-043 also possesses activity against MDR and XDR strains. It inhibits cell wall biosynthesis, and targets the DprE1 (Rv3790) subunit of the enzyme decaprenylphosphoryl-beta-D-ribose 2'-epimerase. BTZ-043 has good oral bioavailability (Figure 23).

##### 3.2.1.6. Tryptanthrin

**Figure 24:**
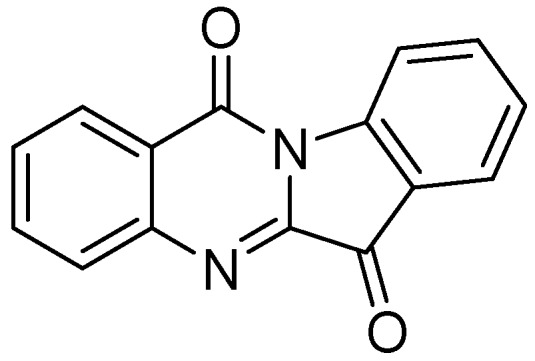
Structure of tryptanthrin.

Tryptanthrin (indolo [2,1-b]quinazolin-6,12-dione), is a natural product that was obtained from a Chinese plant, *Strobilanthes cusia*. It has broad-spectrum biological activities including anti-tuberculosis property. Tryptanthrin demonstrated MIC of 1 μg/mL against *Mtb *in BACTEC assay. It showed MIC values of 0.5–1.0 μg/mL against MDR-TB strains [95]. Preclinical evaluation of tryptanthrin has been conducted [96] (Figure 24).

#### 3.2.2. Molecules in Phase I Clinical Trials

##### AZD-5847

AZD-5847 is an oxazolidinone antibiotic (structure is not disclosed), originally developed for staphylococcal infections. It possesses an MIC_90_ of 1 μg/mL against laboratory *Mtb* strains and clinical isolates resistant to INH, RMP, streptomycin, EMB or OFX [97].

#### 3.2.3. Molecules in Phase II Clinical Trials

##### 3.2.3.1. PNU-100480

**Figure 25:**
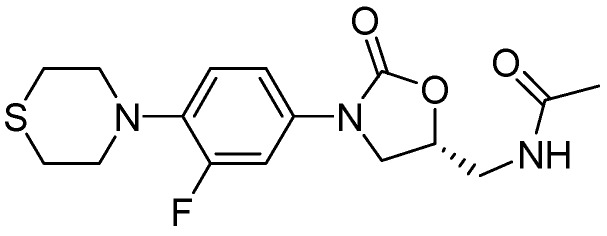
Structure of PNU-100480.

PNU-100480 is a linezolid derivative and is more active (MIC = 0.0625–0.5 μg/mL) than the parent compound and with similar efficacy to that of INH and RMP [98] (Figure 25).

##### 3.2.3.2. LL-3858 or Sudoterb

**Figure 26:**
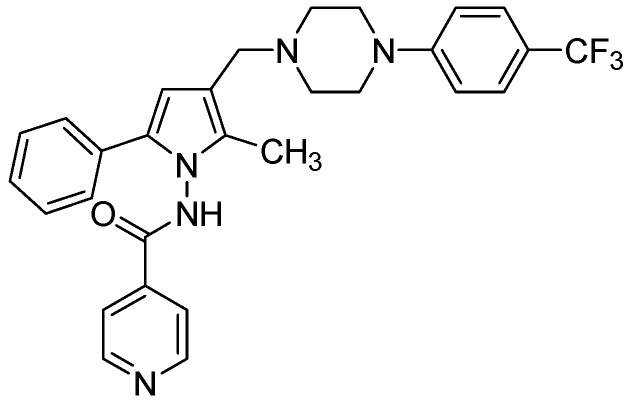
Structure of LL3858.

LL3858 (MIC90 0.25 μg/mL) in combination with current anti-TB drugs, is reported to clear *Mtb* from the lungs and spleens in less time than conventional therapy [99]. The mechanism of action for this class of compounds has not yet been established (Figure 26).

##### 3.2.3.3. SQ-109

**Figure 27:**
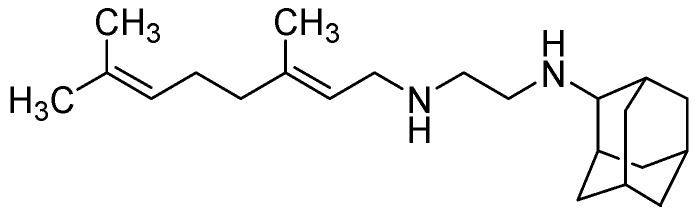
Structure of SQ109.

SQ109 has an MIC = 0.1–0.63 μg/mL [100]. *In vivo* it exhibited 1 to 2.0-log reduction in CFU counts in the lungs and spleens at 25 mg/kg. However, its oral bioavailability was found to be poor (only 4%) [101] (Figure 27).

##### 3.2.3.4. Nitroimidazoles

###### PA-824

**Figure 28:**
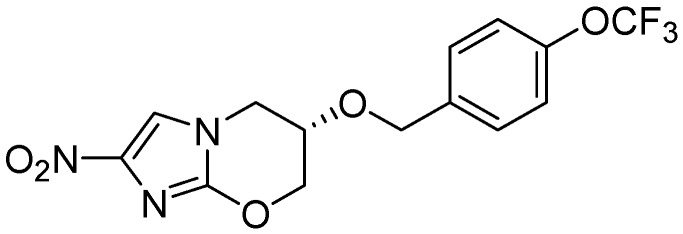
Structure of PA-824.

PA-824 possesses an MIC in the range of 0.015 to 0.25 μg/ml and also retains activity against resistant isolates. It acts by inhibiting the synthesis of protein and cell wall lipids [102]. In a mouse model PA-824 was highly active for latent TB in combination with moxifloxacin [103]. Its minimum bactericidal dose (to reduce the lung CFU count by 99%) was found to be 100 mg/kg/day in murine studies. It is also effective against MDR strains and *Mtb* grown under oxygen depletion [104,105] (Figure 28).

##### 3.2.3.5. OPC-67683 (Delamanid)

**Figure 29:**
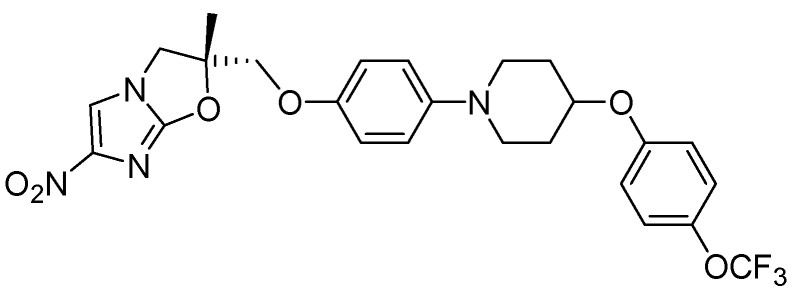
Structure of OPC-67683.

OPC-67683 exhibited an MIC of 0.006 μg/mL [106]. In a mouse model, its efficacy was reported to be superior to existing anti-tuberculosis drugs without any evidence of cross-resistance. The mechanism of action of OPC-67683 is suggested to be similar to PA-824 [107] (Figure 29).

##### 3.2.3.6. TMC-207 or R-207910 or Bedaquiline

**Figure 30:**
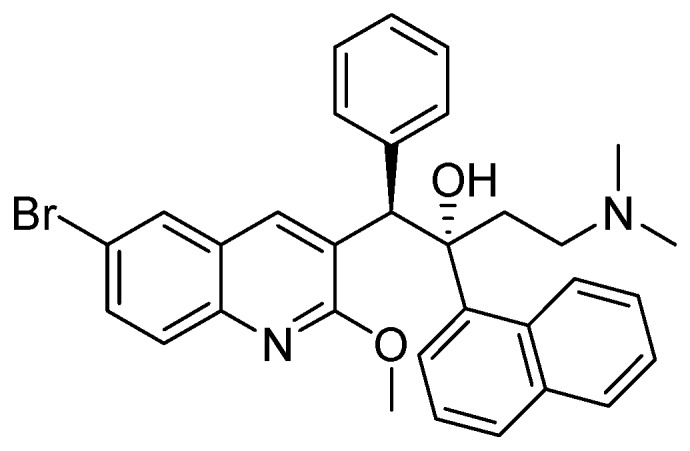
Structure of TMC-207.

The MIC value of TMC-207 ranges from 0.002 to 0.06 µg/mL for drug susceptible and drug resistant (INH, RMP, streptomycin, EMB, PZA and moxifloxacin) strains. It works on the proton pump of ATP synthase [108,109]. In mice, a single dose had bactericidal potency for about eight days. When used as monotherapy, a single dose of TMC-207 was as potent as the triple combination of RMP, INH, and PZA and was more active than RMP alone (Figure 30).

##### 3.2.3.7. Linezolid for the Treatment of MDR-Tuberculosis

**Figure 31:**
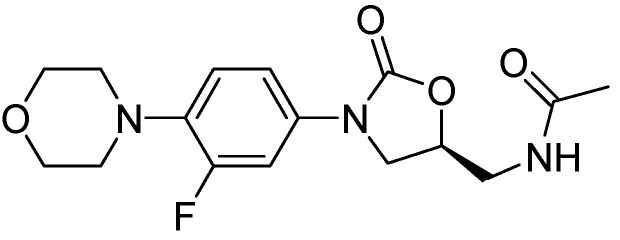
Structure of linezolid.

Linezolid is an approved antibacterial drug (MIC_90_ 1–2 μg/mL) but has not been approved for TB [110]. One of the major concerns for its use as an anti-TB drug is the lack of information on its efficacy [111]. Its long-term use indicated thrombocytopenia, neuropathy and haematopoietic suppression [112] (Figure 31).

##### 3.2.3.8. Rifapentine (TBTC Study)

**Figure 32:**
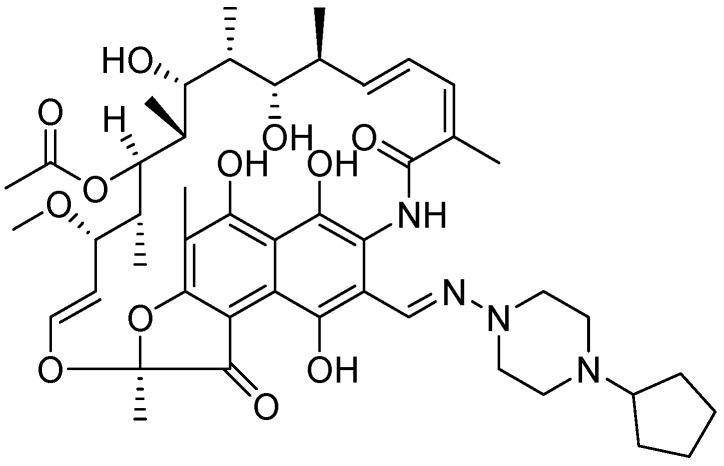
Structure of rifapentine.

Rifapentine is a derivative of rifampicin with an MIC of 0.03 μg/mL [113]. Its mode of action is similar to that of rifampicin [114]. Rifapentine can be used to treat latent TB in combination with either moxifloxacin or INH [103] (Figure 32).

#### 3.2.4. Molecules in Phase III Clincal Trials

##### Fluoroquinolones (Moxifloxacin and Gatifloxacin)

**Figure 33:**
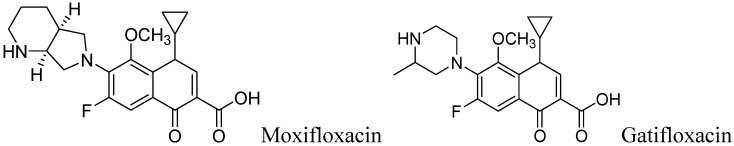
Structures of moxifloxacin and gatifloxacin.

Moxifloxacin, a broad-spectrum antibiotic (400 mg/day dose, MIC of 0.5 μg/mL), is active against both gram-positive and gram-negative bacteria. It displayed early bactericidal activity comparable to INH and rifampin in humans [115,116]. It binds to DNA gyrase and topisomerase IV, which are involved in bacterial replication. Moxifloxacin has no cross-resistance to other antituberculosis drug classes and has been shown to display good activity against MDR strains [117]. However, it has CNS side effects and drug interactions with other fluoroquinolones. Moxifloxacin has not been reported to be safe or effective in children younger than 18 year or in pregnant or lactating women [118]. Nuermberger *et al*. found that substituting moxifloxacin for INH shortens the duration of therapy for active disease much better than does substituting moxifloxacin for EMB [119] (Figure 33).

Gatifloxacin is also a broad-spectrum antibiotic (dosage of 400 mg/day) and has the same mechanism of action as moxifloxacin. It is active against occasionally dividing *Mtb*, but not for dormant bacteria [120]. However, it can cause CNS toxicity and has been associated with increases in insulin levels among diabetics. Like moxifloxacin, it has also not been shown to be safe or effective in children younger than 18 years or in pregnant or lactating women.

## 4. Conclusions

Drug resistance is a critical issue in the treatment of TB. Combined and intensive efforts are required to discover new classes of anti-tuberculosis drugs, otherwise TB could become untreatable in the near future. Currently, several groups/institutions are working together to achieve this goal. These efforts should be continued and intensified to fight this ancient but re-emerging disease. To augment and bolster the development of new drugs for TB, government, private and public authorities need to enhance financial support for research at all levels, and modify regulations to ease the process of evaluation, validation and approval of new drugs. In addition, education and awareness by government, public and private agencies must contribute to preventing the spread of TB and drug resistant MDR or XDR TB.
